# Astragalus affects fecal microbial composition of young hens as determined by 16S rRNA sequencing

**DOI:** 10.1186/s13568-018-0600-9

**Published:** 2018-04-30

**Authors:** Hongxing Qiao, Liheng Zhang, Hongtao Shi, Yuzhen Song, Chuanzhou Bian

**Affiliations:** 1 College of Veterinary Medicine, Henan University of Animal Husbandry and Economy, Longzihu North road No.6, Zhengzhou, 450046 Henan People’s Republic of China; 2 Probiotics Bio-transformation Engineering Technology Research Center of Henan Province, Zhengzhou, 450046 Henan People’s Republic of China; 3 Key Laboratory of Probiotics Fermentation Traditional Chinese Medicine of Zhengzhou city, Zhengzhou, 450046 Henan People’s Republic of China

**Keywords:** Herbal medicine, Microbiota, NGS, *Lactobacillus*, *Romboutsia*

## Abstract

The gut microbiota play important roles in the degradation of chemical compounds of herbal medicines (HMs). However, little information regarding the interplay between HMs and the gut microbiota is available. Thus, the aim of this study was to investigate the composition of the fecal microbiota of young (age, 11 weeks) hens fed a conventional diet containing a crude *Astragalus* (0.5%) additive for 21 days (group A) vs. controls (group B) that were fed only conventional feed. The fecal contents of 14-week-old hens were collected for DNA extraction, and then the V3 and V4 hyper-variable regions of the 16S rRNA gene were amplified and analyzed using high-throughput sequencing technology. A distinctive difference in microbial diversity was observed between the two groups. The microbial composition of hens fed a diet supplemented with *Astragalus* was greater than that of the control group. At the genus level, *Lactobacillus* was more abundant in group A than group B (*p *< 0.05). Importantly, this study is the first to report the observation of a novel *Romboutsia* sp. in the feces of hens. However, *Romboutsia* was less abundant in group A than group B (17.94 vs. 33.98%, respectively, *p *< 0.05). The microbial community differed significantly between the two groups at the genus level, suggesting that *Astragalus* modulates the composition of the fecal microbiota. Based on these differences, these findings provide fresh insights into the application of *Astragalus* in the poultry industry, as well as a better understanding of the interplay between HMs and the gut microbiota.

## Introduction

Poultry meat and eggs are common nutritious and healthy sources of animal protein for human consumption. However, the accumulation of antibiotic residues in chickens and eggs, and the subsequent prevalence of drug-resistant pathogens have received attention worldwide (Wang et al. 2017b). The use of antibiotics can result in gut dysbiosis, diarrhea, and host immune dysregulation (Willing et al. 2011), which results in reduced growth and production (Gao et al. 2017). Recent studies have shown that the composition of the gut microbiota affects various physiological functions of the host, such as nutrient utilization, gut epithelium nourishment, and the development and activity of the gut immune system (Ismail et al. 2009 and Hill et al. 2010).

Herbal medicines (HMs), such as botanical medicines and phytomedicines, have contributed significantly to human and animal health when used to treat disease (Qiu 2007). Moreover, a recent study focusing on the interplay between HMs and the gut microbiota showed that the chemical components of HMs were metabolized by the gut microbiota to generate metabolites that have different bioactivities, which can also mediate the composition of the gut microbiota, restore host homeostasis, and ameliorate gut dysfunction (Chen et al. 2016). A multitude of studies supports the use of HMs, herbal formulas, and phytochemicals, such as Gegen Qinlian Decoction formula (Xu et al. 2015), Qushi Huayu Decotion formula (Yang et al. 2017), Rhizoma coptidis (Xie et al. 2011), and quercetin (Li et al. 2016), to modulate the gut microbiota. Therefore, the gut microbiota plays an important role in the therapeutic efficacy of HMs (Xu et al. 2017).

In China, *Astragalus* is a common HM that contains polysaccharides, saponins, flavonoids, anthraquinones, alkaloids, amino acids, β-sitosterol, and metallic elements (Ibrahim et al. 2013; Li et al. 2009). The major polysaccharides present in *Astragalus* are mannose, d-glucose, d-galactose, xylose, and l-arabinose (Kallon et al. 2013). Our previous studies showed that *Astragalus* fermented by *Bacillus subtilis* using liquid fermentation technology is beneficial to *Astragalus* polysaccharide production (Qiao et al. 2017). The major flavonoids of *Astragalus* are 3-*O*-*β*-d-gluside,2′-hydroxy-3′,4′-dimethoxyisoflavane7-*O*-*β*-d-gluside,7,3′-dihydroxy-4′-methoxyisoflavone,7,3-dimer-capto-4,1-methoxyisoflavone, 3-dimercapto-7,4,1-methoxyisoflavone, and kumatakenin (Lv et al. 2011; Xiao et al. 2004). Moreover, *Astragalus* has anti-inflammatory (Kim et al. 2013), immunostimulatory (Qin et al. 2012), antioxidative (Kim and Yang 2005), and antiviral activities (Sanpha et al. 2013). Therefore, *Astragalus* is used as an additive to the feed of livestock and poultry.

High-throughput, next-generation sequencing (NGS) based on 16S rRNA gene amplicons has the advantage of exploring the more complex aspects of the microbiota in animals. Moreover, studies have reported the composition of the gut microbiota of broiler chickens (Mohd Shaufi et al. 2015), Dagu chickens (Xu et al. 2016), Naked Neck chickens (Park et al. 2016), and egg-laying hens (Videnska et al. 2014). The major phyla in fecal samples of laying hens include Firmicutes (58.8%), Bacteroidetes (22.1%), Proteobacteria (16.9%), Actinobacteria (0.6%), and Fusobacteria (1.4%) (Videnska et al. 2014). Some functional feed additives, such as prebiotics (Rastall and Gibson 2015), probiotics (Gao et al. 2017), phytase (Borda-Molina et al. 2016), and fermented Ginkgo leaves (Zhang et al. 2015), are reported to maintain microbial populations and support the health of the host. NGS has facilitated in-depth studies on the effect of HM on gut microbiota. However, few studies have investigated the microbiota of young hens fed a diet containing *Astragalus*. The results of our previous study showed that both fermented and unfermented *Astragalus* can modulate the intestinal microbiota of broilers (unpublished observations). In the present study, we hypothesized that *Astragalus* supplementation may alter the composition of the fecal microbiota of young laying hens. Using the Illumina MiSeq platform, we attempted to determine whether *Astragalus* is associated with changes in the gut microbiota.

## Materials and methods

### Birds and management

A total of 60 Hy-Line Brown hens (mean age, 11 weeks; mean body weight, 1.32 kg) were assigned randomly to two groups (A and B) of 30 pullets each. For each group, 10 pullets were housed in separate pens (area, > 3000 cm^2^) with ad libitum access to feed and water, a constant room temperature at 16–25 °C, and a 14:10-h light:dark cycle. The hens were checked twice daily by trained staff during the entire 3-week experimental period. All procedures were performed in accordance with the guidelines of the Ministry of Agriculture of China. Hens in group B served as the control group and were fed only conventional feed (Table 1), while those in group A were fed conventional feed supplemented with a crude *Astragalus* (0.5%) additive throughout the experimental period. The animal experiments were conducted in accordance with the Guidelines for the Care and Use of Experimental Animals established and approved by the Laboratory Animal Management Committee of Henan University of Animal Husbandry and Economy.

**Table 1:** Composition of the basic diet (percentage of dried weight)

Ingredient	Control group	Treatment group
Corn	66.70	66.70
Soybean meal	21.70	21.20
Wheat bran	6.20	6.20
Fish meal	2.00	2.00
Dicalcium phosphate	1.10	1.10
Limestone	1.30	1.30
Premix	1.00	1.00
*Astragalus*	0	0.50
Total	100	100

### Preparation of Astragalus

*Astragalus*, the dried root of *Astragalus membranaceus* (Fisch.) *Bge*. var. mongholicus was obtained from a Chinese medicine market (Minxian, Gansu, China) and verified by Dr. Zhang Jing Yu (Henan University of Traditional Chinese Medicine, Zhengzhou, Henan, China). The contents of *Astragalus* polysaccharides, astragalosides, and total flavonoids were 23.420, 0.170, and 4.755 mg/g, respectively. Then, the *Astragalus* root was ground into powder, sieved through a 100-mesh filter, and stored at 25 °C for further use.

### Sample collection and DNA extraction

A total of 27 fecal samples (13 from group A and 14 from group B) were chosen randomly from the hens at the age of 14 weeks. The fecal samples were immediately stored at − 20 °C until DNA extraction. DNA was isolated from 200 mg of feces from each hen using a commercial DNA extraction kit (Tiangen Biotech Corporation, Beijing, China) and quantified using a Qubit 2.0 fluorometer (Invitrogen Corporation, Carlsbad, CA, USA). The quality of the extracted DNA was assessed by 0.8% agarose gel electrophoresis and spectrophotometry (optical density at 260/280 nm). All extracted DNA samples were stored at − 20 °C until further analysis.

### Library preparation and Illumina MiSeq sequencing

NGS library preparations and Illumina MiSeq sequencing were conducted by GENEWIZ, Inc. (Suzhou, China). For the library preparation, a library sequence of the V3 and V4 regions of 16S rRNA was constructed using a 10-ng DNA aliquot isolated from each fecal sample. The V3 and V4 regions were amplified by polymerase chain reaction (PCR) using the following primer pair: forward 5′–CCT ACG GRR BGC ASC AGK VRV GAA T–3′ and reverse 5′–GGA CTA CNY VGG GTW TCT AAT CC–3′. The first-round PCR products were used as templates for a second round of amplicon enrichment by PCR (94 °C for 3 min, followed by 24 cycles at 94 °C for 5 s, 57 °C for 90 s, and 72 °C for 10 s, and a final extension at 72 °C for 5 min). At the same time, indexed adapters were added to the ends of the 16S rDNA amplicons to generate indexed libraries that were ready for downstream NGS on the MiSeq platform. DNA libraries were validated using an Agilent 2100 Bioanalyzer (Agilent Technologies, Palo Alto, CA, USA) and quantified with a Qubit 2.0 Fluorometer. DNA libraries were multiplexed and loaded on an Illumina MiSeq instrument (Illumina, San Diego, CA, USA) according to the manufacturer’s instructions. Sequencing was performed using a 2 × 300 paired-end configuration and image analysis and base calling were conducted with the MiSeq Control Software embedded in the MiSeq instrument. The library used in this study was constructed from a total of 27 DNA samples. The sequences generated in this study have been deposited in the Sequence Read Archive of the National Center for Biotechnology Information (https://www.ncbi.nlm.nih.gov/biosample) under the accession numbers SAMN07135769–7135795.

### Statistical analysis

The QIIME (Quantitative Insights Into Microbial Ecology; ver. 1.9.1) data analysis package was used for 16S rRNA data analysis. The forward and reverse reads were joined and assigned to samples based on barcodes, and truncated by cutting off the barcode and primer sequences. Quality filtering of the joined sequences was performed and sequences that did not fulfill the following criteria were discarded: sequence length < 200 bp, no ambiguous bases, and mean quality score ≥ 20. Then, the sequences were compared with a reference database [the Ribosomal Database Project (RDP) Gold database, 2.2] using the UCHIME algorithm to detect chimeric sequences, which were subsequently removed.

The effective sequences were used in the final analysis. Sequences were grouped into operational taxonomic units (OTUs) using the clustering program VSEARCH (1.9.6) against the SILVA 119 database that was pre-clustered at a 97% sequence identity. The RDP classifier was used to assign a taxonomic category to all the OTUs at a confidence threshold of 0.8. Taxonomic categories at the species level were predicted with the RDP classifier and the SILVA 119 database.

Sequences were rarefied prior to calculating alpha and beta diversity statistics. Alpha diversity indices were calculated in QIIME from rarefied samples using the Shannon and Simpson indexes for diversity, and the Chao1 and ACE indexes for richness. Statistical analysis was performed with the Student’s *t* test using IBM SPSS Statistics 24 (IBM Corp., Armonk, NY, USA) and the results are presented as the mean ± standard deviation. Beta diversity was calculated using weighted and unweighted UniFrac distances, and principal coordinate analysis (PCoA) was performed. An arithmetic mean phylogenetic tree was constructed from the beta diversity distance matrix with an unweighted pair group method. The Student’s *t*-test was employed for analysis of the relative abundance at the phylum and genus levels. A probability (*p*) value of< 0.05 was considered statistically significant. Differences between the two groups were compared using STAMP (2.1.3) analysis.

## Results

### OTU clustering and annotation

**Fig. 1:**
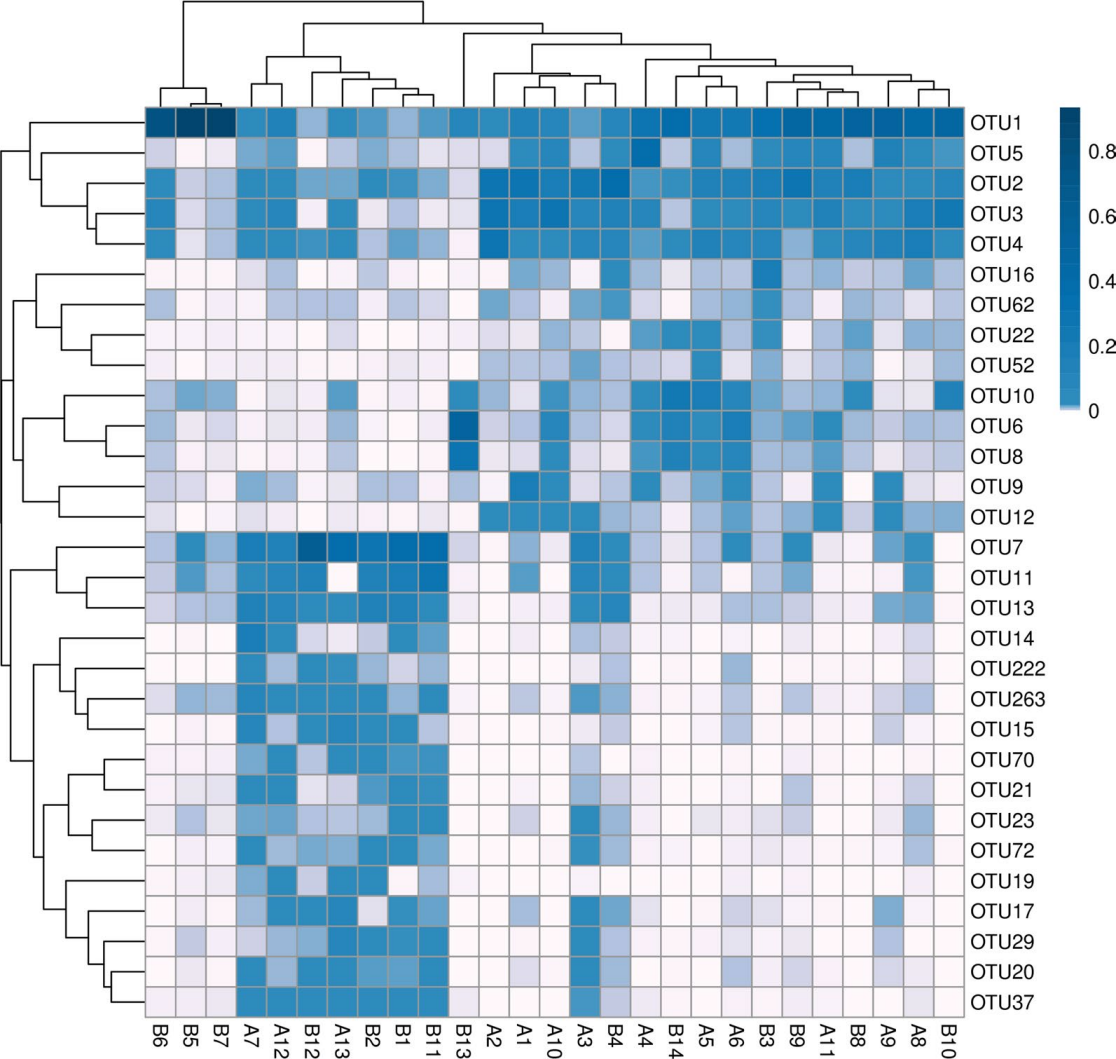
Clustering analysis of the 30 most abundant OTUs in groups A and B. Hens in group A was fed conventional feed supplemented with a crude *Astragalus* (0.5%) additive, while those in group B was fed only conventional feed

**Fig. 2:**
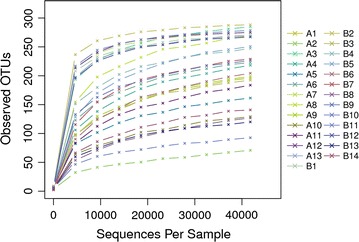
Rarefaction curve sequences showing the complexities of the microbial communities in the fecal samples of 27 young hens. Samples A1–A13 were collected from hens fed conventional feed supplemented with a crude *Astragalus* (0.5%) additive, while samples B1–B14 were collected from hens fed conventional feed

A total of 60 Hy-Line Brown hens at the age of 11 weeks were assigned randomly to group A or B. Hens in group B were fed only conventional feed, while those in group A were fed conventional feed supplemented with 0.5% crude *Astragalus* throughout the entire experimental period. Twenty-seven fecal samples (14 from group A and 13 from group B) were collected. A total of 2,945,166 sequence reads with a median length 450 bp were obtained from all fecal samples. The sequences were further clustered into 317 OTUs using a 97% similarity cut-off. A clustering analysis of 30 OTUs with the highest default abundances showed both similarities and differences between the samples (Fig. 1). For example, the abundance of OTU1 was similar across 27 samples, while there were no differences in OTU2, 3, 4, and 5, except for individual samples (B5, B7, B13). There were some similarities in the OTUs among samples A7, A12, A13, B1, B2, B11, and B12, and dissimilarities among the rest. Using an abundance-based coverage estimator, the Chao1, Simpson’s index Ds, and Shannon’s index H’ identified differences in species richness between the groups (Table 2). The Simpson index of group A was significantly higher (*p *< 0.05) than that of group B, while there were no significant differences in the ACE, Chao1 and Shannon indexed between two groups. The results indicated that species diversity was more abundant in group A than in the group B. The rarefaction curves of the microbiota of 27 samples were sufficiently large to estimate phenotype richness and microbial community diversity at a similarity threshold of 97% (Fig. 2). The rarefaction curves indicated that the sampling effort had sufficient sequence coverage to accurately describe the bacterial composition of each group.

**Table 2:** Diversity estimation of the 16S rRNA gene libraries of the 27 samples from the 16S rRNA sequences

Group	ACE	Chao 1	Shannon	Simpson
A (n = 13)	245.0552 ± 51.5936	248.4307 ± 55.2267	4.0145 ± 1.1526	0.8572 ± 0.0739^a^
B (n = 14)	241.7484 ± 54.5922	247.0312 ± 55.5076	3.2576 ± 1.4677	0.6973 ± 0.0617^b^

Group A = control; Group B = basal diet + 0.5% *Astragalus*

^a,b^Different superscript letters in the same column indicate significant differences

### Microbial beta diversity analysis

**Fig. 3:**
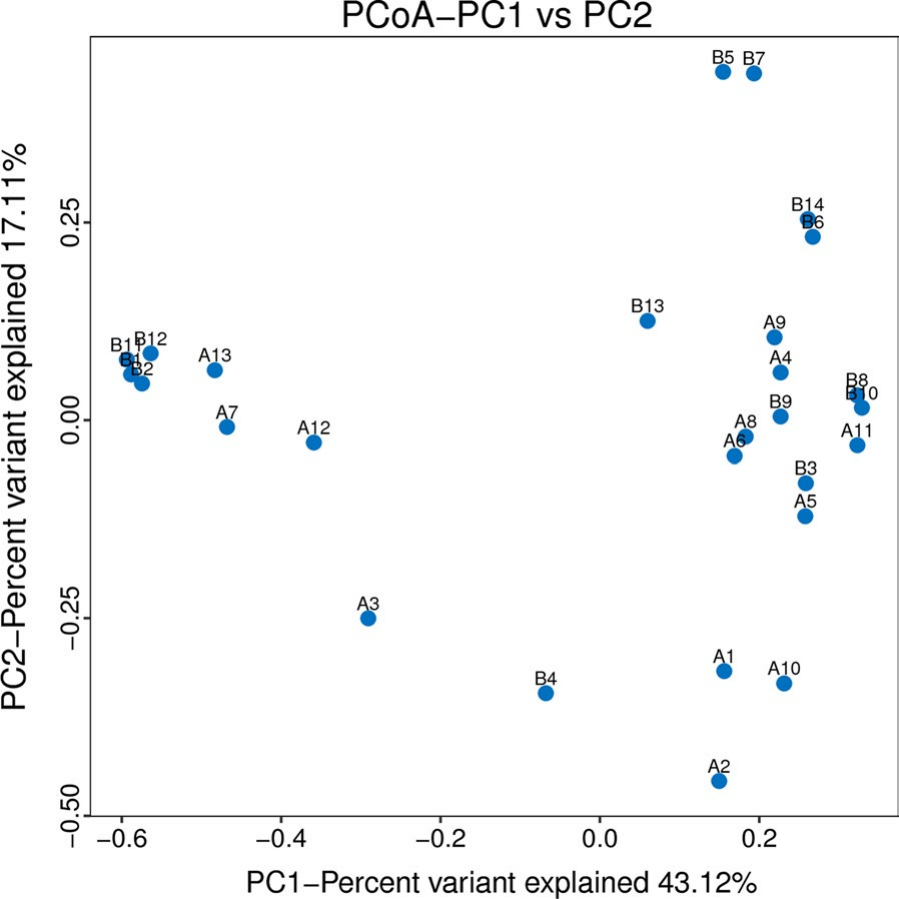
PCoA plot. There were overlaps among four clusters of the 27 samples from groups A and B. Samples A7, A12, A13, B1, B2, B11, and B12 were similar, as were the communities of samples A1, A2, A3, B4, and A10, while the other samples belonged to the same cluster

In a beta diversity analysis, the variations of the microbial community composition in 27 samples were shown in PCoA plots, with PC1 accounting for 43.12% of the total variation and PC2 accounting for 17.11% (Fig. 3). There were overlaps among four clusters and the microbial communities of samples A7, A12, A13, B1, B2, B11, and B12 were similar, as were the communities of samples A1, A2, A3, B4, and A10, while the other samples belonged to the same cluster.

### Bacterial community composition

**Fig. 4:**
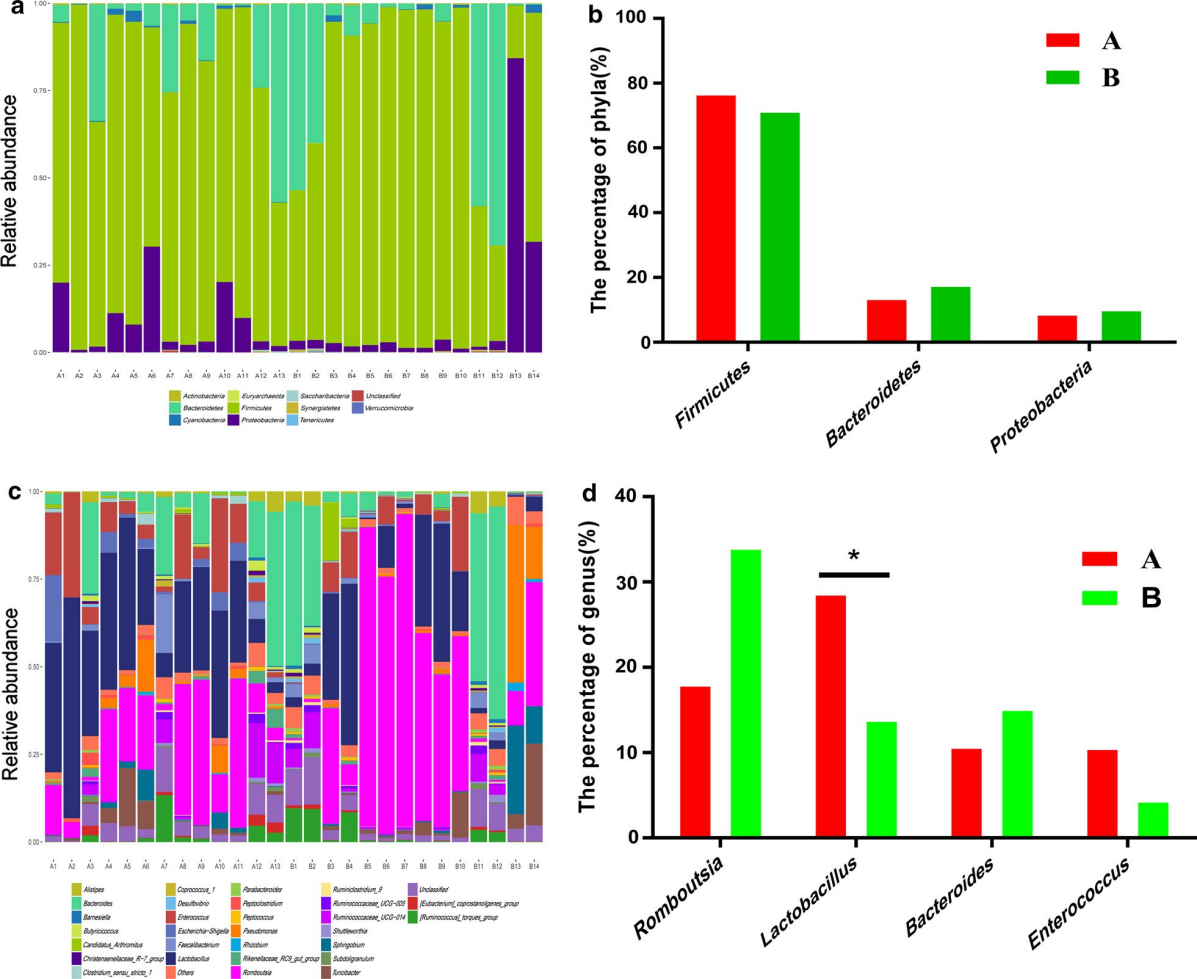
Phylum-level and genus-level analyses of the 27 samples. **a** Overall fecal microbiota composition of the samples at the phylum level in groups A and B. **b** Comparison of the relative abundances of the three main bacterial phyla in groups A and B. **c** Overall fecal microbiota composition of the samples at the genus level in groups A and B. **d** Comparison of the relative abundances of the four main bacterial genus in groups A and B. The relative abundances of *Romboutsia*, *Lactobacillus*, *Bacteroides*, and *Enterococcus* are shown on the *y*-axis. Boxes with one asterisk indicate significant differences between the two groups at *p *<* 0.05* using a *t* test

At the phylum level, a total of 11 dominant phyla were identified in the two groups (Fig. 4a). Most of the sequences in group A were identified as *Firmicutes* (76.71%), *Bacteroidetes* (13.50%), and *Proteobacteria* (8.70%) species, while *Firmicutes* (71.39%), *Bacteroidetes* (17.60%), and *Proteobacteria* (10.01%) species were most abundant in group B (Fig. 4b).

As shown in Fig. 4c, d, at the genus level, the sequences from the 27 samples represented 31 dominate genera in total. *Romboutsia* (17.94%), *Lactobacillus* (28.61%), *Bacteroides* (10.65%), and *Enterococcus* (10.51%) were the most abundant taxa in group A, while *Romboutsia* (33.98%), *Lactobacillus* (13.80%), *Bacteroides* (15.07%), and *Enterococcus* (4.35%) were the most abundant in group B. The differences in the abundances of *Lactobacillus* were significant (*p *< 0.05).

### A community compositional heat map combined with a cluster analysis

**Fig. 5:**
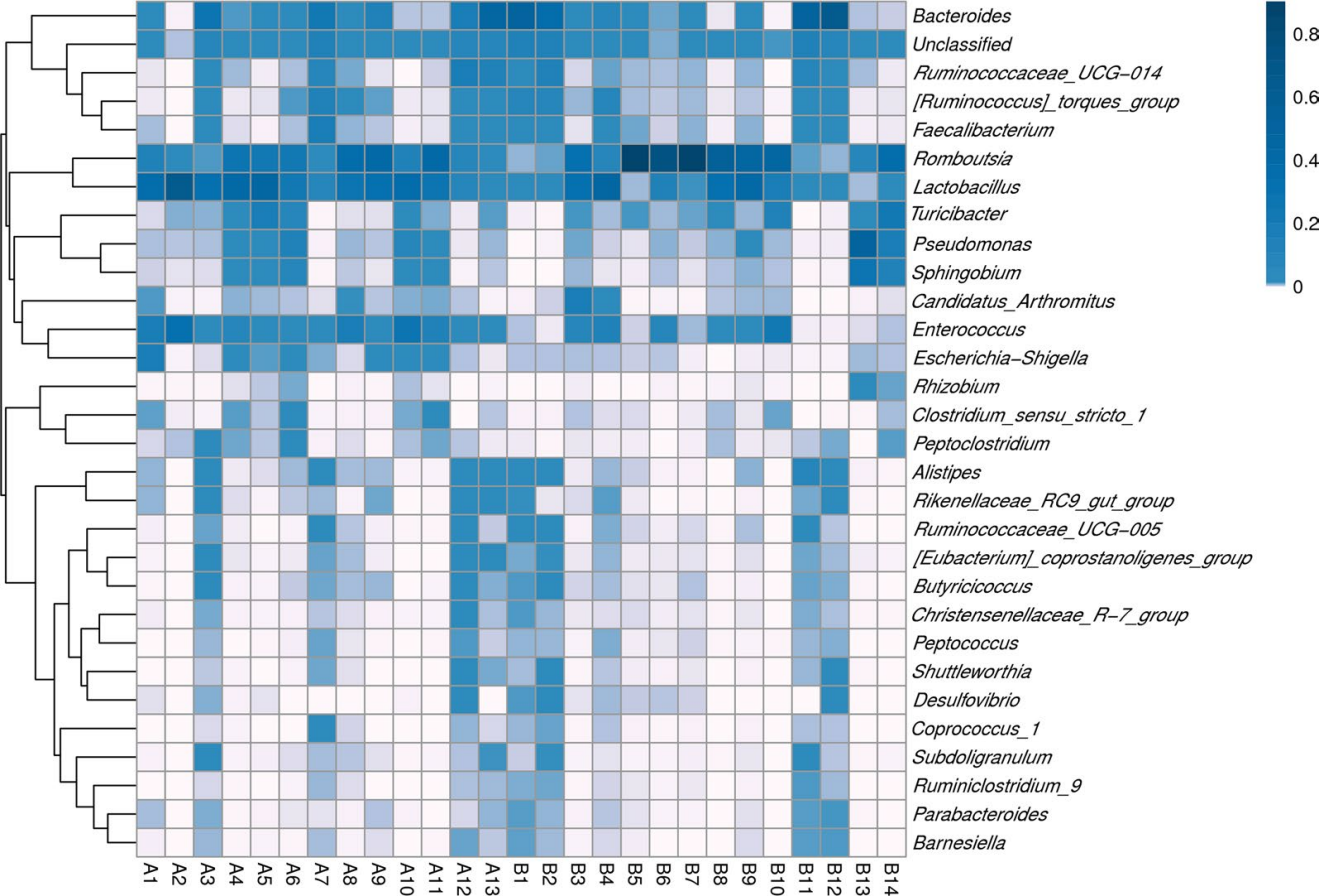
Heat map analysis of the 27 samples. Heat map showing the abundances of the top 30 genera were clustered and plotted using R software. Blue represents genera with higher abundances in the corresponding sample, and white represents genera with lower abundances

As shown in Fig. 5, the top 30 genera in terms of abundance were clustered and plotted using R software. The genera with higher abundances in the corresponding samples are shown in blue and those with lower abundances are shown in white. The populations of *Lactobacillus* and *Romboutsia* increased remarkably between groups A and B. Moreover, *Romboutsia* was more abundant in group B than group A. The reason for this difference may be partially attributed to the different pH of the feed fed to groups A and B (pH 4.96 vs. 4.71, respectively).

### Statistical analysis of taxonomic and functional profiles (STAMP)

**Fig. 6:**
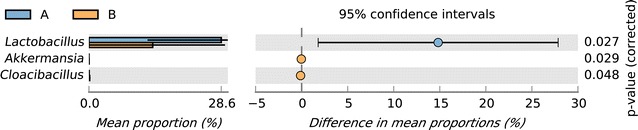
The STAMP analysis of groups A and B. *Lactobacillus* was more abundant in group A than group B

Differences between the two groups at the genus level were compared using STAMP (v2.1.3) software and the Welch’s *t*-test. As shown in Fig. 6, the abundance of *Lactobacillus* was significantly (*p *< 0.05) greater in group A than group B. However, differences in the abundances of other genera were not significant.

## Discussion

Although many studies have investigated the gut microbiota composition of poultry, very few have assessed the interactions between HMs and gut microbes. In recent years, the potential application of HMs in aquatic animals and rat models has been increasingly explored. A recent study showed that Yu-Ping-Feng (Jade screen) powder (Chinese parsnip root, *Astragalus membranaceus* and *Atractylodes macrocephala Koidz*) can regulate the intestinal microbiota of fish (Wu et al. 2018), the combination of Gancao-Gansui impacted the gut microbiota diversity of the rat (Yu et al. 2018), and *Salvia miltiorrhiza Bge.* modulated the microbiota imbalance in diabetic mice (Gu et al. 2017). Previous studies have shown that fermented Ginkgo leaves contributed to the microbial ecology in the gut of broiler chicks (Zhang et al. 2015), while fermented pine needles (*Pinus ponderosa*) improved antioxidant status and growth performance in broilers (Wu et al. 2015) and improved meat quality and lipid metabolism in broilers (Cao et al. 2012). The principle of HMs supplementation of feed lies in maintaining or restoring the balance or enhancing the ability of immune defenses (Guo et al. 2004). Therefore, the aim of the present study was to compare the gut microbiota composition of young laying hens fed a diet supplemented with or without *Astragalus* supplement.

In this study, at the phylum level, *Firmicutes*, *Bacteroidetes*, and *Proteobacteria* were the most common phyla in the poultry fecal samples, which is consistent with earlier findings by Danzeisen et al. (2011) and Yeoman et al. (2012). Our results illustrate that *Firmicutes* were the dominant phylum (> 50%) in young laying hens, an observation that is consistent with previous reports (Mohd Shaufi et al. 2015; Danzeisen et al. 2011). Previous studies have also suggested that a lower abundance of *Bacteroidetes* spp. was associated with increased body weight (Arumugam et al. 2011; Ley et al. 2006). The results of the present study showed that the abundance of *Bacteroidetes* app. was lower in hens fed a diet supplemented with *Astragalus*, as compared with the control group, as *Astragalus* produces two kinds of polysaccharides that are common carbohydrates (Xu et al. 2008).

Numerous studies have also shown that *Bacteroidetes* aid in the host metabolism of polysaccharides, which improves nutrient utilization (Bäckhed et al. 2004) and host immunity (Hooper 2004; Stappenbek et al. 2002), as well as maintenance of the intestinal microecological balance (Hooper et al. 2001; Sears 2005). Animals are unable to digest and utilize complex polysaccharides in feedstuff in the absence of microbial fermentation (Vrize et al. 2010). In addition, our results differ from those of Singh et al. (2012) who showed that *Proteobacteria* was the most dominant phylum. The abundances of *Proteobacteria*, which include a wide range of pathogens, such as *Escherichia*, *Salmonella*, *Helicobacter*, and *Vibrio* spp., were slightly lower in group A than in group B, indicating that the abundance of opportunistic pathogens might decrease in response to supplementation with *Astragalus.* Therefore, the addition of *Astragalus* to feed may be beneficial for modulating the gut microbiota of young hens.

At the genus level, *Romboutsia*, *Lactobacillus*, *Bacteroides*, and *Enterococcus* were identified as the dominant species of the fecal microbiome. STAMP analysis results revealed that *Lactobacillus* was more abundant in group A than group B (28.61% vs. 13.80%, respectively, *p *< 0.05), in agreement with the reports by Stanley et al. (2012) and Gong et al. (2007). Moreover, a recent study showed that the abundance of *Lactobacillus* was highly related to the feeding efficiency of the host (Yan et al. 2017). *Lactobacillus* is an important probiotic that promotes the gut health of both humans and animals via the production of various short-chain fatty acids. In addition, the results of a rodent study showed that non-digestible fermentable carbohydrates and fibers can enhance the growth of specific beneficial gut bacteria (Bindels et al. 2015). It is reported that feeding different metabolite combinations produced by *Lactobacillus plantarum* increased the abundance of fecal lactic acid bacteria and reduced the population of *Enterobacteriaceae* in the gut of laying hens (Loh et al. 2014). Therefore, feed supplementation with *Astragalus* results in an increase in the abundance of *Lactobacillus*, which is thought to be indicative of the health of hens. Moreover, in this study, the abundance of *Bacteroides* was lower in group A than group B. *Bacteroides*, which include the genera *Bacteroides*, *Prevotella* and *Xylanibacter*, are known to be efficient degraders of dietary fiber (Simpson and Campbell 2015). *Bacteroides* are thought to play fundamental roles in the breakdown of complex polysaccharides, suggesting that *Bacteroides* may be involved in the metabolism of *Astragalus* polysaccharides.

To the best of our knowledge, this is the first study of a novel *Romboutsia* genus (group A 17.94%; group B 33.98%) in fecal samples of hens, as members of the *Romboutsia* genus have never before been detected in the digestive tract of hens. *Romboutsia* spp. have been identified in the human gut (Ricaboni et al. 2016), lake sediment (Wang et al. 2015), and the rat gastrointestinal tract (Gerritsen et al. 2014). The results of the present study demonstrated that *Astragalus* supplementation enriched the abundance of *Lactobacillus* and reduced the abundance of *Romboutsia* spp. Therefore, we speculate there is a competitive exclusion relationship between the abundance of *Romboutsia* spp. and *Lactobacillus* genera, although well-designed experiments focusing on bacterial interactions are required to verify these results.

It is well know that *Astragalus* polysaccharides mainly consist of glucose and xylose. *Romboutsia* is able to utilize glucose, but not xylose (Wang et al. 2015). However, the effect of *Romboutsia* metabolism on the quantity of these monosaccharides remains unclear, thus further studies are warranted. In a future study, we plan to isolate and characterize the functions of *Romboutsia* spp.

Although fecal samples, instead of the cecal samples, were used to study the microbiome in this study, a previous study showed similarities in the microbial communities, but quantitative differences between cecum and fecal samples (Stanley et al. 2015). Moreover, as compared to cecal contents, fecal samples are easier to collect for analysis of the productivity, health, and wellbeing of chickens. However, sampling from cecum to other intestinal tract sections requires sacrificing of the hens. In contrast, fecal sampling allows repeated sampling over time and does not require sacrifice (Stanley et al. 2015). The results of the present study demonstrated that structural changes to the fecal microbiota are induced by *Astragalus.*

A previous study reported that HMs can alter the composition of the intestinal microbial community while being metabolized (Xu et al. 2017). Single HMs, HM formulas, and even individual compounds of HMs are capable of affecting the gut microbiota. Therefore, HMs may either promote or inhibit the gut microbiota. Similarly, some probiotics, such as *Lactobacillus plantarum*, have been shown to modulate fecal parameters in chickens (Gao et al. 2017), and *Lactobacillus johnsonii* has been shown to improve the gut microbiota in broiler chickens (Wang et al. 2017a). In our study, the addition of single *Astragalus* also modulated fecal microbiota. On the one hand, this might be due to the main bioactive constituents of *Astragalus*, such as polyphenols, which have anti-inflammatory and immunomodulatory effects that promote the abundances of some gut bacteria. On the other hand, it might result from interactions among gut microbiota after *Astragalus* feeding.

In conclusion, *Astragalus* is rich in fiber and polysaccharides that can be fermented and converted into short-chain fatty acids, which are beneficial to health and play a vital role in the modulation of fecal microbiota, although deciphering the underlying mechanism will require further research.

## Abbreviations

HMs: herbal medicines
NGS: high-throughput, next-generation sequencing
OTUs: operational taxonomic units
PCR: polymerase chain reaction
PCoA: principal coordinate analysis
STAMP: statistical analysis of taxonomic and functional profiles

## References

[CR1] Arumugam M, Raws J, Pelletier E, Le Paslier D, Yamada T, Mende DR, Fernandes GR, Tap J, Bruls T, Batto JM, Bertalan M, Borruel N, Casellas F, Fernandez L, Gautier L, Hansen T, Hattori M, Hayashi T, Kleerebezem M, Kurokawa K, Leclerc M, Levenez F, Manichanh C, Nielsen HB, Nielsen T, Pons N, Poulain J, Qin JJ, Sicheritz-Ponten T, Tims S, Torrents D, Ugarte E, Zoetendal EG, Wang J, Guarner F, Pedersen OM, de Vos W, Brunak S, Dore J, Consortium M, Weissenbach J, Dusko-Ehrlich S, Bork P (2011). Enterotype of the human gut microbiome. Nature.

[CR2] Bäckhed F, Ding H, Wang T, Hooper LV, Koh GY, Nagy A, Semenkovich CF, Gordon JI (2004). The gut microbiota as an environmental factor that regulates fat storage. Proc Natl Acad Sci USA.

[CR3] Bindels LB, Delzenne NM, Cani PD, Walter J (2015). Towards a more comprehensive concept for prebiotics. Nat Rev Gastroenterol Hepatol..

[CR4] Borda-Molina D, Vital M, Sommerfeld V, Rodehutscord M, Camarinha-Silva A (2016). Insights into Broiler’s gut microbiota fed with phosphorus, calcium, and phytase supplemented diets. Front Microbiol..

[CR5] Cao FL, Zhang XH, Yu WW, Zhao LG, Wang T (2012). Effect of feeding fermented *Ginkgo biloba* leaves on growth performance meat quality, and lipid metabolism broilers. Poult Sci.

[CR6] Chen F, Wen Q, Jiang J, Li HL, Tan YF, Li YH, Zeng NK (2016). Could the gut microbiota reconcile the oral bioavailability conundrum of traditional herbs?. J Ethnopharmacol.

[CR7] Danzeisen JK, Kim HB, Isaacson RE, Tu ZJ, Johnson TJ (2011). Modulation of the chicken cecal microbiome and metagenome in response to anticoccidial and growth promoter treatment. PLoS ONE.

[CR8] Gao PF, Hou QC, Kwok Lai-Yu, Huo DX, Feng SZ, Zhang HP (2017). Effects of feeding *Lactobacillus plantarum* P-8 on the faecal microbiota of broiler chickens exposed to lincomycin. Sci Bull..

[CR9] Gerritsen J, Fuentes S, Grievink W, Niftrik LV, Tindall BJ, Timmerman HM, Rijkers GT, Smidt H (2014). Characterization of *Romboutsia ilealis* gen. nov., sp. nov., isolated from the gastro-intestinal tract of a rat, and proposal for the reclassification of five closely related members of the genus *Clostridium* into the genera *Romboutsia* gen. nov., *Intestinibacter* gen. nov., *Terrisporobacter* gen. nov. and *Asaccharospora* gen. nov. Int J Syst Evol Microbiol.

[CR10] Gong J, Si W, Forster RJ, Huang R, Yu H, Yin Y, Yang C, Han Y (2007). 16S rRNA gene-based analysis of mucosa-associated bacterial community and phylogeny in the chicken gastrointestinal tracts: from crops to ceca. FEMS Microbiol Ecol.

[CR11] Gu JF, Su SL, Guo JM, Zhu Y, Zhao M, Duan JA (2017). The aerial parts of *Salvia miltiorrhiza Bge*. strengthen intestinal barrier and modulate gut microbiota imbalance in streptozocin-induced diabetic mice. J Funct Foods..

[CR12] Guo FC, Williams BA, Kwakkel RP, Li HS, Li XP, Luo JY, Li WK, Verstegen MWA (2004). Effects of mushroom and herb polysaccharides, as alternatives for an antibiotic, on the cecal microbial ecosystem in broiler chickens. Poult Sci.

[CR13] Hill DA, Hoffmann C, Abt MG, Du Y, Kobuley D, Kirna TJ, Bushmanb FD, Artisa D (2010). Metagenomic analysis reveal antibiotic-induced temporal and spatial changes in intestinal microbiota with associated alterations in immune cell homeostasis. Mucosal Immunol.

[CR14] Hooper LV (2004). Bacterial contribution to mammalian gut development. Trends Microbiol.

[CR15] Hooper LV, Wong MH, Thelin A, Hansson L, Falk PG, Gordon JI (2001). Molecular analysis of commensal host-microbial relationship in the intestinal. Science.

[CR16] Ibrahim LF, Marzouk MM, Hussein SR, Kauashty SA, Mahmoud K, Saleh NAM (2013). Flavonoid constituents and biological screening of *Astragalus* bombycinus Boiss. Nat Prod Res.

[CR17] Ismail AS, Behrendt CL, Hooper LV (2009). Reciprocal interactions between commensal bacteria and gamma delta intraepithelial lymphocytes during mucosal injury. J Immunol..

[CR18] Kallon S, Li X, Ji J, Chen C, Xi Q, Chang S, Xue C, Ma J, Xie Q, Zhang Y (2013). *Astragalus* polysaccharide enhances immunity and inhibits H9N2 avian influenza virus in vitro and in vivo. J Anim Sci Biotechnol.

[CR20] Kim EJ, Yang KS (2005). Anti lipid peroxidative activity of *Radix Astragali ceus*. Yakhak Hoechi.

[CR21] Kim JH, Kim MH, Yang G, Huh Y, Kim SH, Yang WM (2013). Effects of topical application of *Radix Astragalus* on allergic dermatitis. J Immunopharmacol Immunotoxicol.

[CR22] Ley RE, Turnbaugh PJ, Klein S, Gordon JI (2006). Microbial ecology: human gut microbes associated with obesity. Nature.

[CR23] Li SP, Zhao XJ, Wang JY (2009). Synergy of *Astragalus* polysaccharides and probiotics (*Lactobaciilus* and *Bacillus* cereus) on immunity and intestinal microbiota in chicks. Poult Sci.

[CR24] Li Y, Yao J, Han C, Yang J, Chaudhry MT, Wang S, Liu H, Yin Y (2016). Quercetin, inflammation and immunity. Nutrients.

[CR25] Loh TC, Choe DW, Foo HL, Sazili AQ, Bejo MH (2014). Effects of feeding different postbiotic metabolite combinations produced by Lactobacillus plantarum strains on egg quality and production performance, faecal parameters and plasma cholesterol in laying hens. BMC Vet Res.

[CR26] Lv YW, Hu W, Wang YL, Huang LF, He YB, Xie XZ (2011). Identification and determination of flavonoida in astragali radix by high performance liquid chromatography coupled with DAD and ESI-MS detection. Molecules.

[CR27] Mohd Shaufi MA, Sieo CC, Chong CW, Gan HM, Ho YW (2015). Deciphering chicken gut microbial dyamics based on high-throughput 16S rRNA metagenomics analyses. Gut Pathog.

[CR28] Park SH, Lee SI, Ricke SC (2016). Microbial populations in naked neck chicken ceca raised on pasture flock fed with commercial yeast cell wall prebiotics via an Illumina MiSeq Platform. PLoS ONE.

[CR29] Qiao HX, Shi HT, Zhang Z, Jiang YL, Bian CZ (2017). Characteristics of Bacillus subtilis HNMY-13 and HNMY-15 strains in aflatoxin B1 degradation and *Astragalus* bio-transformation. Afr J Biotechnol.

[CR30] Qin Q, Niu J, Wang Z, Xu W, Qiao Z, Gu Y (2012). *Astragalus* membaranceus inhibits inflammation via phospho-p38 mitogen-activated protein kinase (MAPK) and nuclear factor (NF)-ĸB pathways in advanced glycation end product-stimulated macrophages. Int J Mol Sci.

[CR31] Qiu J (2007). Traditional medicine—a culture in the balance. Nature.

[CR32] Rastall RA, Gibson GR (2015). Recent development in prebiotics to selectively impact beneficial microbes and promote intestinal health. Curr opin biotechnol.

[CR33] Ricaboni D, Mailhe M, Khelaifia S, Raoult D, Million M (2016). Romboutsia timonensis, a new species isolated from human gut. New Microbes New Infec..

[CR034] Sanpha K, Li XY, Ji J, Chen CY, Xi QY, Chang S, Xue QY, Ma J, Xie Q, Zhang Y (2013). *Astragalus* polysaccharide enhances immunity and inhibits H9N2 avian influenza virus in vitro and in vivo. J Anim Sci Biotechnol.

[CR34] Sears CL (2005). A dynamic partnership: celebrating our gut flora. Anaerobe.

[CR35] Simpson HL, Campbell BJ (2015). Review article: dietary fibre-microbiota interactions. Aliment Pharmacol Ther.

[CR36] Singh KM, Shah T, Deshpande S, Jakhesara S, Koringa P, Rank DN, Joshi CG (2012). High through put 16S rRNA gene-based pyrosequencing analysis of the fecal microbiota of high FCR and low FCR broiler growers. Mol Biol Rep.

[CR37] Stanley D, Denman SE, Hughes RJ, Geier MS, Crowley TM, Chen H, Haring VR, Moore RJ (2012). Intestinal microbiota associated with differential feed conversion efficiency in chickens. Appl Microbiol Biotechnol.

[CR38] Stanley D, Geier MS, Chen HL, Hughes RJ, Moore RJ (2015). Comparison of fecal and cecal microbiotas reveals qualitative similarities but quantitative differences. BMC Microbiol.

[CR39] Stappenbek TS, Hooper LV, Gordon JI (2002). Development regulation of intestinal angiogenesis by indigenous microbes via Paneth cells. Proc Natl Acad Sci USA.

[CR40] Videnska P, Rahman MM, Faldynova M, Babak V, Matulova ME, Prukner-Radocic E, Krizek I, Smole-Mozina S, Kovac J, Szmolka A, Nagy B, Sedlar K, Cejkova D, Rychlik I (2014). Characterization of egg laying hen and broiler fecal microbota in poultry farms in Croatia, Czech republic, Hungary and Slovenia. Plos ONE.

[CR41] Vrize A, Holleman F, Zoetenal EG, de Vos WM, Hoekstra JB, Nieuwdorp M (2010). The environment within: how gut microbiota may influence metabolism and body composition. Diabetologia.

[CR42] Wang YW, Song JL, Zhai Y, Zhang C, Gerritsen J, Wang HM, Chen XL, Li YT, Zhao BQ, Zhao B, Ruan ZY (2015). *Romboutsia sedimentorum* sp. nov., isolated from an alkaline-saline lake sediment and emended description of the genus *Romboutsia*. Int J Syst Evol Microbiol.

[CR43] Wang HS, Ni XQ, Qing XD, Zeng D, Luo M, Liu L, Li GY, Pan KC, Jing B (2017). Live probiotic *Lactobacillus johnsonii* BS15 promotes growth performance and lowers fat deposition by improving lipid metabolism, intestinal development, and gut microflora in broilers. Front Microbiol.

[CR44] Wang Y, Zhang RM, Li JY, Wu ZW, Yin WJ, Schwarz Stefam, Tyrrell JM, Zheng Y, Wang S, Shen Z, Liu Z, Liu J, Lei L, Li M, Zhang Q, Wu C, Zhang Q, Wu Y, Walsh TR, Shen J (2017). Comprehensive resistome analysis reveals the prevalence of NDM and MCR-1 in Chinese poultry production. Nat Microbiol.

[CR45] Willing BP, Russell SL, Finalay BB (2011). Shifting the balance: antibiotic effects on host-microbiota mutualism. Nat Rev Microbiol.

[CR46] Wu QJ, Wang ZB, Wang GY, Li YX, Qi YX (2015). Effects of feed supplemented with fermented pine needles (Pinus ponderosa) on growth performance and antioxidant status in broilers. Poult Sci.

[CR47] Wu ZB, Gatesoupe FJ, Li TT, Wang XH, Zhang QQ, Feng DY, Feng YQ, Chen H, Li AH (2018). Significant improvement of intestinal microbiota of gibel carp (*Carassius auratus gibelio*) after traditional Chinese medicine feeding. J Appl Microbiol.

[CR48] Xiao HB, Krucker M, Albert K, Liang XM (2004). Determination and identification of isoflavonoids in *Radix astragali* by matrix solid-phase dispersion extraction and high-performance liquid chromatography with photodiode array and mass spectrometric detection. J Chromatogr A.

[CR49] Xie W, Gu D, Li J, Cui K, Zhang Y (2011). Effect and action mechanisms of berberine and Rhizoma coptidis on gut microbes and obesity in high-fat die-fed C57BL/6J mice. PLoS ONE.

[CR50] Xu DJ, Xia Q, Wang JJ, Wang PP (2008). Molecular weight and monosaccharide composition of *Astragalus* polysaccharides. Molecules.

[CR51] Xu J, Lian F, Zhao L, Zhao Y, Chen X, Zhang X, Guo Y, Zhang C, Zhou Q, Xue Z, Pang X, Zhao L, Tong X (2015). Structural modulation of gut microbiota during alleviation of type 2 diabetes with a Chinese herbal formula. ISME J.

[CR52] Xu YH, Yang HX, Zhang LL, Su TH, Shi DH, Xiao HD, Tian Y (2016). High-throughput sequencing technology to reveal the composition and function of cecal microbiota in Dgu chicken. BMC Microbiol.

[CR53] Xu J, Chen HB, Li SL (2017). Understanding the molecular mechanisms of the interplay between Herbal medicine and gut microbiota. Med Res Rev.

[CR54] Yan W, Sun CJ, Yuan JW, Yang N (2017). Gut metagenomic analysis reveals prominent role of *lactobacillus* and cecal microbiota in chicken feed efficiency. Sci Rep.

[CR55] Yang Y, Chen G, Yang Q, Ye J, Cai X, Tsering P, Cheng X, Hu C, Zhang S, Cao P (2017). Gut microbiota drives the attenuation of dextran sulphate sodium-induces colitis by Huangqin decoction. Oncotarget.

[CR56] Yeoman CJ, Chia N, Jeraldo P, Sipos M, Goldenfeld ND, White BA (2012). The microbiome of the chicken gastrointestinal tract. Anim Health Res Rev.

[CR57] Yu YG, Guo JM, Tao WW, Liu P, Shang EX, Zhu ZH, Fan XH, Shen J, Hua YQ, Zhu KY, Tang YP, Duan JA (2018). Gancao-Gansui combination impacts gut microbiota diversity and related metabolic functions. J Ethnopharmacol.

[CR58] Zhang X, Sun Z, Cao F, Ahmad H, Yang X, Zhao L, Wang T (2015). Effects of dietary supplementation with fermented Ginkgo leaves on antioxidant capacity, intestinal morphology and microbial ecology in broiler chicks. Br Poult Sci.

